# Supplementary material for: Diffusion and Viscosity in Mixed Protein Solutions
Source: J Phys Chem B. 2024 Nov 19;128(47):11676–93. doi: 10.1021/acs.jpcb.4c06877 (PMC11613455; doi:10.1021/acs.jpcb.4c06877)
Supplement: Supplementary file 1 — jp4c06877_si_001.pdf [file jp4c06877_si_001.pdf]

SUPPLEMENTARY INFORMATION

for

Diffusion and Viscosity in Mixed Protein Solutions

Spencer Wozniak[1] and Michael Feig[1,*]

[1]Department of Biochemistry and Molecular Biology, Michigan State University, East Lansing, MI 48824, USA

*Corresponding author
Michael Feig
603 Wilson Road, Room 218 BCH
East Lansing, MI 48824, USA
mfeiglab@gmail.com
+1-517-432-7439

Tables S1-S15
Figures S1-S15
Supplementary References

Table S1. Amino acid sequences of simulated systems

Protein	Uniprot	Residues *(numbering in full sequence)*	Sequence
SH3	Q08012	1-59	MEAIAKHDFS ATADDELSFR KGQILKILNM EDDSNWYRAE LDGKEGLIPS NYIEMKNHD
GB1	P06654	228-282	MQYKLILNGK TLKGETTTEA VDAATAEKVF KQYANDNGVD GEWTYDDATK TFTVTE
LYS	P00698	19-147	KVFGRCELAA AMKRHGLDNY RGYSLGNWVC AAKFESNFNT QATNRNTDGS TDYGILQINS RWWCNDGRTP GSRNLCNIPC SALLSSDITA SVNCAKKIVS DGNGMNAWVA WRNRCKGTDV QAWIRGCRL
BSA	P02769	27-607	HKSEIAHRFK DLGEEHFKGL VLIAFSQYLQ QCPFDEHVKL VNELTEFAKT CVADESHAGC EKSLHTLFGD ELCKVASLRE TYGDMADCCE KQEPERNECF LSHKDDSPDL PKLKPDPNTL CDEFKADEKK FWGKYLYEIA RRHPYFYAPE LLYYANKYNG VFQECCQAED KGACLLPKIE TMREKVLTSS ARQRLRCASI QKFGERALKA WSVARLSQKF PKAEFVEVTK LVTDLTKVHK ECCHGDLLEC ADDRADLAKY ICDNQDTISS KLKECCDKPL LEKSHCIAEV EKDAIPENLP PLTADFAEDK DVCKNYQEAK DAFLGSFLYE YSRRHPEYAV SVLLRLAKEY EATLEECCAK DDPHACYSTV FDKLKHLVDE PQNLIKQNCD QFEKLGEYGF QNALIVRYTR KVPQVSTPTL VEVSRSLGKV GTRCCTKPES ERMPCTEDYL SLILNRLCVL HEKTPVSEKV TKCCTESLVN RRPCFSALTP DETYVPKAFD EKLFTFHADI CTLPDTEKQI KKQTALVELL KHKPKATEEQ LKTVMENFVA FVDKCCAADD KEACFAVEGP KLVVSTQTAL A
OVA	P01012	2-386	GSIGAASMEF CFDVFKELKV HHANENIFYC PIAIMSALAM VYLGAKDSTR TQINKVVRFD KLPGFGDSIE AQCGTSVNVH SSLRDILNQI TKPNDVYSFS LASRLYAEER YPILPEYLQC VKELYRGGLE PINFQTAADQ ARELINSWVE SQTNGIIRNV LQPSSVDSQT AMVLVNAIVF KGLWEKAFKD EDTQAMPFRV TEQESKPVQM MYQIGLFRVA SMASEKMKIL ELPFASGTMS MLVLLPDEVS GLEQLESIIN FEKLTEWTSS NVMEERKIKV YLPRMKMEEK YNLTSVLMAM GITDVFSSSA NLSGISSAES LKISQAVHAA HAEINEAGRE VVGSAEAGVD AASVSEEFRA DHPFLFCIKH IATNAVLFFG RCVSP

Deviations from native sequence are indicated in red.

Table S2. Molecular dynamics simulation details

System	Replicates	Length[a] [μs]	Average box size [Å]	Atoms	Ensemble	Temperature [K]	Pressure [atm]
sh3_gb1_50	3	2	98.86	98,235	NPT	298	1
sh3_gb1_100	3	2	98.49	97,663	NPT	298	1
sh3_gb1_300	3	2	96.35	93,629	NPT	298	1
sh3_lys_50	3	2	109.90	134,678	NPT	298	1
sh3_lys_200	3	2	98.84	99,241	NPT	298	1
sh3_lys_300	3	2	96.99	94,572	NPT	298	1
sh3_bsa_50	3	1	160.06	415,939	NPT	298	1
sh3_bsa_100	3	2	126.72	207,398	NPT	298	1
sh3_bsa_200	3	2	100.03	103,019	NPT	298	1
sh3_bsa_300	3	2	109.79	137,415	NPT	298	1
sh3_ova_50	3	1.2	138.19	267,604	NPT	298	1
sh3_ova_300	3	2	108.25	131,428	NPT	298	1
sh3_urea_50	3	2	96.00	89,777	NPT	298	1
sh3_suc_300	3	2	92.82	84,254	NPT	298	1
sh3	3	1	61.57	23,693	NPT	298	1
gb1	3	1	59.52	21,405	NPT	298	1
lys	3	1	67.50	31,227	NPT	298	1
bsa	3	1	116.27	159,611	NPT	298	1
ova	3	1	108.01	128,282	NPT	298	1
urea	3	1	32.15	3,356	NPT	298	1
suc	3	1	36.98	5,113	NPT	298	1
wat_salt[b]			49.276[c]	11,938	NVT	298	1
wat[b]			49.288[d]	11,982	NVT	298	1

[a]common trajectory length in all three replicates used for analysis
[b]water systems were run for viscosity calculations and to extract translational diffusion
[c]fixed to achieve density of 1.005 g/L
[d]fixed to achieve density of 0.997 g/L

Table S3. Experimental reference structures

Protein	PDB	Method	Resolution
SH3	2A37[1]	NMR	
GB1	2LGI[2]	NMR	
LYS	3WL2	X-ray	0.95 Å
BSA	3V03[3]	X-ray	2.70 Å
OVA	1OVA[4]	X-ray	1.95 Å

Table S4. Coordinate root mean square deviations of proteins

System	Average[a] Cα-RMSD [Å][b]				
	**SH3**	**GB1**	**Lysozyme**	**BSA**	**Ovalbumin**
sh3_gb1_50	2.15 *(0.03)*	1.05 *(0.02)*			
sh3_gb1_100	2.20 *(0.14)*	1.15 *(0.02)*			
sh3_gb1_300	2.10 *(0.11)*	1.12 *(0.03)*			
sh3_lys_50	2.24 *(0.13)*		2.70 *(0.05)*		
sh3_lys_200	2.21 *(0.16)*		2.72 *(0.11)*		
sh3_lys_300	2.11 *(0.08)*		2.53 *(0.07)*		
sh3_bsa_50	2.23 *(0.03)*			3.02 *(0.20)*	
sh3_bsa_100	2.19 *(0.12)*			2.86 *(0.23)*	
sh3_bsa_200	2.51 *(0.34)*			3.11 *(0.04)*	
sh3_bsa_300	2.16 *(0.12)*			3.41 *(0.14)*	
sh3_ova_50	2.65 *(0.54)*				2.94 *(0.14)*
sh3_ova_300	2.07 *(0.15)*				3.08 *(0.08)*
sh3_urea_50	2.45 *(0.09)*				
sh3_suc_300	2.20 *(0.19)*				
sh3	2.14 *(0.07)*				
gb1		1.04 *(0.13)*			
lys			1.57 *(0.28)*		
bsa				3.54 *(0.06)*	
ova					3.28 *(0.09)*

[a]omitting the first 400 ns of each trajectory; standard errors given in parentheses were estimated from variations between replicate simulations
[b]with respect to the PDB structures given in **Table S3** after optimal superposition

Table S5. SH3-Crowder contact survival

System	SH3-crowder					
	τ_1 [ns]	τ_2 [ns]	τ_3 [ns]	w_1	w_2	w_3[b]
sh3_gb1_50	69.1	3.5	0.11	0.77	0.16	0.07
sh3_gb1_100	57.6	2.7	0.08	0.80	0.13	0.07
sh3_gb1_300	112.6	1.6	0.02	0.55	0.19	0.26
sh3_lys_50	541.0	3.2	0.08	0.95	0.03	0.02
sh3_lys_200	332.9	2.2	0.05	0.74	0.08	0.18
sh3_lys_300	336.7	1.7	0.04	0.75	0.06	0.19
sh3_bsa_50	47.4	1.0	0.22	0.64	0.36	0.00
sh3_bsa_100	>10 μs[a]	9.6	0.29	0.70	0.22	0.08
sh3_bsa_200	599.9	3.5	0.08	0.85	0.07	0.08
sh3_bsa_300	503.8	2.0	0.07	0.90	0.03	0.07
sh3_ova_50	1074.4	0.80	0.06	0.99	0.01	0.00
sh3_ova_300	650.4	1.2	0.10	0.77	0.05	0.18
sh3_urea_50	75.5	0.68	0.08	0.04	0.52	0.44
sh3_suc_300	11.5	2.5	0.15	0.33	0.44	0.23

from triple-exponential fits (**Eq. 5**) to combined survival functions from all replicates up to 20 ns
[a]exact results from the numerical fits are not reported when the fitted time scales exceed 10 μs
[b]w_3=1.0-w_2-w_1

Table S6. Crowder-Crowder contact survival

System	Crowder-crowder					
	**τ_1 [ns]**	**τ_2 [ns]**	**τ_3 [ns]**	**w_1**	**w_2**	**w_3[b]**
sh3_gb1_50	70.1	5.0	0.18	0.70	0.22	0.08
sh3_gb1_100	63.7	3.5	0.10	0.70	0.20	0.10
sh3_gb1_300	92.2	1.7	0.02	0.57	0.15	0.28
sh3_lys_50	320.0	8.3	0.20	0.91	0.07	0.03
sh3_lys_200	204.4	1.6	0.08	0.71	0.11	0.18
sh3_lys_300	312.4	1.5	0.05	0.66	0.09	0.25
sh3_bsa_50	-[c]					
sh3_bsa_100	>10 µs[a]	2483	0.31	0.53	0.47	0.00
sh3_bsa_200	>10 µs[a]	-	-	1.00	0.00	0.00
sh3_bsa_300	679.9	1.6	0.05	0.86	0.03	0.11
sh3_ova_50	-[d]					
sh3_ova_300	>10 µs[a]	-	-	1.0	0.00	0.00

from triple-exponential fits (**Eq. 5**) to combined survival functions from all replicates up to 20 ns
[a]exact results from the numerical fits are not reported when the fitted time scales exceed 10 µs
[b]w_3=1.0-w_2-w_1
[c]contacts persisted for the entire trajectory
[d]only one dimer present in system

Table S7. Viscosity estimates from simulation

System	Viscosity[a] η [cP]	rel. viscosity η/η_0[c]	rel. viscosity η/η_0[d]
sh3_gb1_50[b]	0.476 *(0.003)*	1.425	1.373
sh3_gb1_100	0.621 *(0.012)*	1.859	1.791
sh3_gb1_300[b]	1.985*(0.002)*	5.943	5.725
sh3_lys_50[b]	0.458 *(0.002)*	1.371	1.321
sh3_lys_200	1.103 *(0.025)*	3.302	3.181
sh3_lys_300[b]	1.733 *(0.022)*	5.189	5.000
sh3_bsa_50[b]	0.472 *(0.002)*	1.413	1.361
sh3_bsa_100	0.599 *(0.009)*	1.793	1.727
sh3_bsa_200	0.987 *(0.015)*	2.955	2.847
sh3_bsa_300[b]	1.868 *(0.024)*	5.593	5.388
sh3_ova_50[b]	0.463 *(0.004)*	1.386	1.335
sh3_ova_300[b]	1.828 *(0.016)*	5.473	5.273
sh3_urea_50	0.421 *(0.003)*	1.260	1.214
sh3_suc_300	1.182 *(0.012)*	3.539	3.409
sh3	0.424 *(0.003)*	1.259	1.213
gb1	0.425 *(0.004)*	1.272	1.225
lys	0.459 *(0.003)*	1.374	1.324
bsa	0.495 *(0.003)*	1.482	1.428
ova	0.549 *(0.007)*	1.644	1.584
urea	0.364 *(0.004)*	1.090	1.050
suc	0.376 *(0.003)*	1.126	1.085
wat_salt	0.347 *(0.003)*	1.038	1.0
wat	0.334 *(0.006)*	1.0	0.963

[a]averages with τ_{max}=100 ps from 150 runs of 1 ns each unless noted otherwise; standard errors were obtained from dividing the total number of runs into 10 blocks consisting of 15 runs each.
[b]averages over 150 runs of 2 ns each
[c]relative to the viscosity of pure water
[d]relative to the calculated viscosity of water with 150 mM salt

Table S8. Fitting coefficients for experimental and simulated viscosity data

Protein	Collloid model (Eq. 20)			Mooney (Eq. 21)			
	fit range	b	χ^2	fit range	S	K	χ^2
lysozyme	0-250 g/L	38.2	0.02	0-400 g/L	2.78	3.01	0.05
BSA	0-250 g/L	93.1	0.05	0-400 g/L	5.67	1.67	0.09
ovalbumin	0-250 g/L	48.2	0.18	0-400 g/L	4.19	2.18	0.02
MD proteins	0-250 g/L	63.4	0.016	0-250 g/L	6.38	0.38	0.007

Table S9. Translational diffusion coefficients from simulations and experiments

**Protein**	**System**	**Translational Diffusion** [Å^2/ns]				
		$\mathbf{D_{t,PBC}}^{a}$	$\mathbf{D_{t,TIP3P}}^{b}$	$\mathbf{D_{t,MD}}^{c}$	$\mathbf{D_{t,exp}}$	$\mathbf{D_{t,HYDROPRO}}^{5}$
SH3	sh3	14.79 *(1.92)*	42.01 *(1.91)*	15.77 *(0.72)*	15[6]	15.03
	sh3_gb1_50	15.40 *(0.30)*	28.19 *(0.30)*	10.58 *(0.11)*	13[6]	
	sh3_gb1_100	8.94 *(1.04)*	18.74 *(1.04)*	7.03 *(0.39)*	8.4[6]	
	sh3_gb1_300	0.35 *(0.15)*	3.46 *(0.15)*	1.30 *(0.05)*		
	sh3_lys_50	8.89 *(0.52)*	20.96 *(0.52)*	7.87 *(0.20)*	7.9[6]	
	sh3_lys_200	1.02 *(0.08)*	6.57 *(0.08)*	2.47 *(0.03)*		
	sh3_lys_300	0.27 *(0.01)*	3.86 *(0.01)*	1.45 *(0.00)*		
	sh3_bsa_50	23.48 *(1.29)*	31.60 *(1.29)*	11.86 *(0.50)*	13[6]	
	sh3_bsa_100	12.50 *(1.98)*	20.54 *(1.98)*	7.71 *(0.77)*	10[6]	
	sh3_bsa_200	1.95 *(0.23)*	8.08 *(0.23)*	3.03 *(0.09)*	4.3[6]	
	sh3_bsa_300	0.34 *(0.08)*	3.30 *(0.08)*	1.24 *(0.03)*		
	sh3_ova_50	13.28 *(1.68)*	22.91 *(1.68)*	8.60 *(0.65)*	11[6]	
	sh3_ova_300	0.47 *(0.15)*	3.54 *(0.15)*	1.33 *(0.06)*		
	sh3_urea_50	21.25 *(0.97)*	36.21 *(0.97)*	13.59 *(0.37)*	15[6]	
	sh3_suc_300	5.31 *(0.03)*	10.81 *(0.03)*	4.06 *(0.01)*	5.6[6]	
GB1	gb1	16.23 *(1.23)*	44.36 *(1.25)*	16.65 *(0.47)*		15.56
	sh3_gb1_50	16.74 *(0.52)*	29.54 *(0.52)*	11.09 *(0.19)*		
	sh3_gb1_100	8.79 *(0.33)*	18.61 *(0.33)*	6.98 *(0.13)*		
	sh3_gb1_300	0.33 *(0.02)*	3.43 *(0.02)*	1.29 *(0.01)*		
Lysozyme	lys	9.93 *(0.92)*	34.23 *(0.92)*	12.85 *(0.35)*	12.85[7]<br>10.3 (@20g/L)[8]<br>10.8 (@14g/L)[9]<br>10.8 (@15g/L)[10]	12.26
	sh3_lys_50	8.75 *(0.47)*	20.68 *(0.47)*	7.76 *(0.18)*	9.1[10]	
	sh3_lys_200	0.95 *(0.03)*	6.42 *(0.03)*	2.41 *(0.01)*		
	sh3_lys_300	0.28 *(0.01)*	3.83 *(0.01)*	1.44 *(0.00)*		
BSA	bsa	5.00 *(0.43)*	19.19 *(0.43)*	7.20 *(0.16)*	6.2[11], 6.3[12]	6.83
	sh3_bsa_50	4.39 *(0.10)*	12.27 *(0.10)*	4.60 *(0.04)*		
	sh3_bsa_100	3.13 *(0.10)*	10.79 *(0.10)*	4.05 *(0.04)*		
	sh3_bsa_200	1.12 *(0.02)*	6.78 *(0.02)*	2.54 *(0.01)*		
	sh3_bsa_300	0.21 *(0.00)*	2.99 *(0.00)*	1.12 *(0.00)*		
Ovalbumin	ova	3.09 *(0.04)*	18.51 *(0.04)*	6.95 *(0.02)*	8.0[13]<br>6.0 (@20 g/L)[10]	6.61
	sh3_ova_50	4.63 *(0.32)*	13.77 *(0.32)*	5.17 *(0.12)*	5[10]	
	sh3_ova_300	0.28 *(0.00)*	3.10 *(0.00)*	1.16 *(0.00)*		

[a]estimated directly from MSD curves according to **Eq. 10** over 2-10 ns
[b]corrected for periodic box artefacts according to **Eq. 11**
[c]corrected for underestimated viscosity with TIP3P water model according to **Eq. 13**

Table S10. Rotational diffusion coefficients from simulations via Wong et al.[14]

Protein	System	τ_1 [ns]	τ_2 [ns]	w	τ_c[a] [ns]	$D_{r,PBC}$[b] [1/µs]	$D_{r,TIP3P}$[c] [1/µs]
SH3	sh3	1.4		1.00	1.41 *(0.03)*	118.0 *(3.0)*	126.3 *(3.0)*
	sh3_gb1_50	7.5	1.8	0.38	2.61 *(0.16)*	64.3 *(3.9)*	65.7 *(3.9)*
	sh3_gb1_100	16.1	2.2	0.62	4.91 *(1.09)*	36.9 *(7.3)*	38.0 *(7.3)*
	sh3_gb1_300	634	1.3	0.96	33.34 *(6.29)*	5.47 *(1.25)*	5.83 *(1.25)*
	sh3_lys_50	15.4	2.4	0.80	7.69 *(1.14)*	22.9 *(3.9)*	24.0 *(3.9)*
	sh3_lys_200	561	1.4	0.96	41.75 *(10.86)*	4.52 *(1.03)*	5.16 *(1.03)*
	sh3_lys_300	1773	0.6	0.99	51.78 *(10.34)*	3.58 *(0.89)*	4.01 *(0.89)*
	sh3_bsa_50	17.2	1.3	0.18	1.62 *(0.13)*	104.1 *(7.6)*	104.5 *(7.6)*
	sh3_bsa_100	24.8	1.4	0.49	2.89 *(0.86)*	67.2 *(16.1)*	67.7 *(16.1)*
	sh3_bsa_200	96.7	1.6	0.94	21.38 *(2.53)*	8.05 *(1.08)*	8.74 *(1.08)*
	sh3_bsa_300	399	1.5	0.95	30.48 *(4.82)*	5.74 *(0.86)*	6.01 *(0.86)*
	sh3_ova_50	21.1	0.9	0.75	3.53 *(0.36)*	48.3 *(5.4)*	48.8 *(5.4)*
	sh3_ova_300	437	1.1	0.96	35.50 *(8.35)*	5.47 *(1.66)*	5.77 *(1.65)*
	sh3_urea_50	3.7	1.4	0.06	1.50 *(0.01)*	111.4 *(1.1)*	113.3 *(1.1)*
	sh3_suc_300	6.5	3.1	0.69	6.27 *(0.18)*	26.6 *(0.8)*	27.3 *(0.8)*
GB1	gb1	1.2		1.0	1.17 *(0.02)*	142.6 *(3.0)*	151.8 *(3.0)*
	sh3_gb1_50	6.6	1.4	0.44	2.10 *(0.11)*	79.8 *(4.3)*	81.3 *(4.3)*
	sh3_gb1_100	17.7	1.9	0.66	4.67 *(0.47)*	36.4 *(3.4)*	37.5 *(3.4)*
	sh3_gb1_300	274	1.6	0.96	35.6 *(3.8)*	4.78 *(0.47)*	5.15 *(0.47)*
Lysozyme	lys	2.9		1.0	2.87 *(0.04)*	58.1 *(0.8)*	64.4 *(0.8)*
	sh3_lys_50	15.3	3.4	0.82	9.34 *(1.18)*	18.4 *(2.2)*	19.5 *(2.2)*
	sh3_lys_200	461	1.6	0.97	53.5 *(7.3)*	3.25 *(0.48)*	3.89 *(0.48)*
	sh3_lys_300	1024	0.9	0.99	65.3 *(1.2)*	2.55 *(0.05)*	2.99 *(0.85)*
BSA	bsa	20.6		1.0	20.6 *(2.6)*	8.34 *(1.00)*	9.58 *(1.00)*
	sh3_bsa_50	47.9	20.0	0.67	38.6 *(3.8)*	4.40 *(0.41)*	4.76 *(0.41)*
	sh3_bsa_100	55.1	93.1	0.81	49.9 *(5.0)*	3.42 *(0.38)*	3.98 *(0.38)*
	sh3_bsa_200	232	3.2	0.99	113.5 *(11.2)*	1.50 *(0.16)*	2.19 *(0.16)*
	sh3_bsa_300	3329	1.5	0.99	246.9 *(66.8)*	0.81 *(0.26)*	1.09 *(0.26)*
Ovalbumin	ova	22.8		1.0	22.8 (2.5)	7.48 *(0.82)*	9.03 *(0.82)*
	sh3_ova_50	29.2	2.9	0.87	21.5 *(3.0)*	8.10 *(1.27)*	8.67 *(1.27)*
	sh3_ova_300	2538	1.0	1.00	239.8 *(36.8)*	0.73 *(0.12)*	1.03 *(0.12)*

[a]not corrected for PBC
[b]estimated from fitted rotational autocorrelation functions according to **Eqs. 14-16**
[c]corrected for periodic box artefacts according to **Eq. 18**

Table S11. Rotational diffusion coefficients from simulations via Linke *et al.*[15]

Protein	System	D_{r2}/D_{r1}	D_{r3}/D_{r1}	$D_{r,PBC}$ [1/μs]	$D_{r,TIP3P}$[a] [1/μs]
SH3	sh3	0.87 *(0.02)*	0.69 *(0.04)*	115.8 *(1.6)*	124.2 *(1.6)*
	sh3_gb1_50	0.84 *(0.02)*	0.74 *(0.02)*	57.5 *(6.1)*	59.0 *(6.1)*
	sh3_gb1_100	0.91 *(0.05)*	0.75 *(0.07)*	28.1 *(5.2)*	29.2 *(5.2)*
	sh3_gb1_300	0.83 *(0.08)*	0.70 *(0.08)*	4.53 *(0.85)*	4.89 *(0.84)*
	sh3_lys_50	0.84 *(0.04)*	0.64 *(0.04)*	18.4 *(2.8)*	19.5 *(2.8)*
	sh3_lys_200	0.79 *(0.07)*	0.68 *(0.05)*	2.90 *(0.65)*	3.54 *(0.65)*
	sh3_lys_300	0.88 *(0.09)*	0.75 *(0.10)*	1.86 *(0.45)*	2.29 *(0.45)*
	sh3_bsa_50	0.88 *(0.06)*	0.68 *(0.01)*	90.3 *(10.6)*	90.7 *(10.6)*
	sh3_bsa_100	0.86 *(0.06)*	0.77 *(0.03)*	48.0 *(13.2)*	48.6 *(13.2)*
	sh3_bsa_200	0.71 *(0.13)*	0.53 *(0.10)*	4.82 *(0.75)*	5.51 *(0.75)*
	sh3_bsa_300	0.82 *(0.07)*	0.66 *(0.05)*	3.66 *(0.64)*	3.94 *(0.64)*
	sh3_ova_50	0.85 *(0.05)*	0.73 *(0.04)*	25.4 *(10.0)*	26.0 *(10.0)*
	sh3_ova_300	0.71 *(0.03)*	0.50 *(0.06)*	3.23 *(0.91)*	3.52 *(0.91)*
	sh3_urea_50	0.88 *(0.02)*	0.79 *(0.05)*	108.3 *(5.3)*	110.1 *(5.3)*
	sh3_suc_300	0.83 *(0.03)*	0.74 *(0.02)*	24.9 *(0.9)*	25.6 *(0.9)*
GB1	gb1	0.74 *(0.07)*	0.62 *(0.05)*	144.7 *(2.9)*	154.0 *(3.0)*
	sh3_gb1_50	0.78 *(0.02)*	0.69 *(0.02)*	66.6 *(3.7)*	68.1 *(3.7)*
	sh3_gb1_100	0.78 *(0.02)*	0.67 *(0.02)*	28.0 *(2.1)*	29.1 *(2.1)*
	sh3_gb1_300	0.74 *(0.01)*	0.61 *(0.01)*	4.29 *(0.13)*	4.65 *(0.13)*
Lysozyme	lys	0.73 *(0.11)*	0.56 *(0.06)*	56.0 *(1.6)*	62.3 *(1.6)*
	sh3_lys_50	0.72 *(0.03)*	0.57 *(0.02)*	15.8 *(1.7)*	16.9 *(1.7)*
	sh3_lys_200	0.73 *(0.02)*	0.60 *(0.02)*	2.14 *(0.19)*	2.79 *(0.19)*
	sh3_lys_300	0.77 *(0.03)*	0.64 *(0.03)*	1.59 *(0.07)*	2.02 *(0.07)*
BSA	bsa	0.76 *(0.04)*	0.52 *(0.03)*	8.17 *(0.74)*	9.41 *(0.74)*
	sh3_bsa_50	0.59 *(0.02)*	0.45 *(0.02)*	3.41 *(0.04)*	3.76 *(0.04)*
	sh3_bsa_100	0.51 *(0.03)*	0.45 *(0.02)*	3.01 *(0.03)*	3.57 *(0.03)*
	sh3_bsa_200	0.52 *(0.09)*	0.39 *(0.10)*	1.19 *(0.06)*	1.88 *(0.06)*
	sh3_bsa_300	0.88 *(0.02)*	0.20 *(0.03)*	1.26 *(0.03)*	1.54 *(0.03)*
Ovalbumin	ova	0.84 *(0.05)*	0.63 *(0.04)*	7.36 *(0.59)*	8.90 *(0.59)*
	sh3_ova_50	0.70 *(0.04)*	0.59 *(0.03)*	6.53 *(0.13)*	7.09 *(0.13)*
	sh3_ova_300	0.45 *(0.09)*	0.15 *(0.02)*	1.21 *(0.04)*	1.51 *(0.04)*

[a]corrected for periodic box artefacts according to **Eq. 18**

Table S12. Rotational diffusion coefficients from simulations and experiments

Protein	System	$D_{r,MD}$[a] [1/µs] Wong *et al.*	$D_{r,MD}$[a] [1/µs] Linke *et al.*	$D_{r,exp}$[b] [1/µs]	$D_{r,HYDROPRO}$ [1/µs]
SH3	sh3	47.41 *(1.11)*	46.61 *(0.59)*	45.0 *(1.2)*[6]	41.3
	sh3_gb1_50	24.66 *(1.47)*	22.13 *(2.30)*	37.0 *(0.8)*[6]	
	sh3_gb1_100	14.25 *(2.72)*	10.95 *(1.96)*	25.3 *(0.4)*[6]	
	sh3_gb1_300	2.19 *(0.47)*	1.84 *(0.32)*		
	sh3_lys_50	9.00 *(1.48)*	7.33 *(1.03)*	11.1 *(0.7)*[6]	
	sh3_lys_200	1.94 *(0.38)*	1.33 *(0.24)*		
	sh3_lys_300	1.50 *(0.34)*	0.86 *(0.17)*		
	sh3_bsa_50	39.21 *(2.87)*	34.02 *(3.98)*	39.7 *(0.9)*[6]	
	sh3_bsa_100	25.42 *(6.05)*	18.23 *(4.95)*	29.8 *(0.5)*[6]	
	sh3_bsa_200	3.28 *(0.41)*	2.07 *(0.28)*	15.2 *(1.3)*[6]	
	sh3_bsa_300	2.26 *(0.32)*	1.48 *(0.24)*		
	sh3_ova_50	18.32 *(2.02)*	9.75 *(3.76)*	13.9 *(1.1)*[6]	
	sh3_ova_300	2.16 *(0.62)*	1.32 *(0.34)*		
	sh3_urea_50	42.51 *(0.40)*	41.33 *(2.00)*	42.7 *(1.1)*[6]	
	sh3_suc_300	10.26 *(0.28)*	9.60 *(0.33)*	16.5 *(0.3)*[6]	
GB1	gb1	56.98 *(1.13)*	57.77 *(1.11)*	51.8[16, 17] (@ 10 g/L, pH=5.3)	48.2
	sh3_gb1_50	30.50 *(1.60)*	25.54 *(1.40)*		
	sh3_gb1_100	14.07 *(1.29)*	10.93 *(0.80)*		
	sh3_gb1_300	1.93 *(0.18)*	1.75 *(0.05)*		
Lysozyme	lys	24.17 *(0.31)*	23.37 *(0.61)*	19.2[18] (@ 80 g/L, pH=4, no salt) 9.5[18] (@ 79 g/L, pH=4, 0.3 M NaCl) 7.6[18] (@ 78 g/L, pH=9, no salt 24.2[19] (@ 50 g/L, pH=3.8, no salt)	23.1
	sh3_lys_50	7.33 *(0.83)*	6.34 *(0.64)*		
	sh3_lys_200	1.46 *(0.18)*	1.04 *(0.07)*		
	sh3_lys_300	1.12 *(0.02)*	0.76 *(0.03)*		
BSA	bsa	3.60 *(0.38)*	3.53 *(0.28)*	3.4[20] (@ 3 g/L, pH=8, no salt)	3.74
	sh3_bsa_50	1.78 *(0.15)*	1.41 *(0.02)*		
	sh3_bsa_100	1.49 *(0.14)*	1.34 *(0.01)*		
	sh3_bsa_200	0.82 *(0.06)*	0.70 *(0.02)*		
	sh3_bsa_300	0.41 *(0.10)*	0.58 *(0.01)*		
Ovalbumin	ova	3.39 *(0.31)*	3.34 *(0.22)*		3.46
	sh3_ova_50	3.25 *(0.48)*	2.66 *(0.05)*		
	sh3_ova_300	0.39 *(0.05)*	0.57 *(0.01)*		

[a]corrected for underestimated viscosity with TIP3P water model according to **Eq. 19**
[b]obtained from experimental values of τ_c via $D_r=1/(6\tau_c)$

Table S13. Hydrodynamic radii according to Stokes-Einstein

**Protein**	**System**	**R_h** [Å] **from $D_{t,TIP3P}$**[a]	**R_h** [Å] **from $D_{r,TIP3P}$**[b] Wong *et al.*	**R_h** [Å] **from $D_{r,TIP3P}$**[b] Linke *et al.*
SH3	sh3	15.03 *(0.65)*	15.52 *(0.12)*	15.60 *(0.07)*
	sh3_gb1_50	16.30 *(0.18)*	17.40 *(0.34)*	18.11 *(0.68)*
	sh3_gb1_100	18.87 *(1.08)*	19.40 *(1.27)*	21.12 *(1.25)*
	sh3_gb1_300	31.92 *(1.28)*	24.63 *(1.59)*	25.95 *(1.36)*
	sh3_lys_50	22.75 *(0.56)*	24.88 *(1.25)*	26.57 *(1.21)*
	sh3_lys_200	30.12 *(0.36)*	31.25 *(2.27)*	35.24 *(2.05)*
	sh3_lys_300	32.58 *(0.07)*	29.24 *(1.94)*	35.08 *(2.07)*
	sh3_bsa_50	14.68 *(0.58)*	14.95 *(0.38)*	15.75 *(0.67)*
	sh3_bsa_100	18.11 *(1.89)*	16.43 *(1.56)*	18.55 *(2.03)*
	sh3_bsa_200	27.40 *(0.79)*	26.83 *(1.03)*	31.36 *(1.41)*
	sh3_bsa_300	35.48 *(0.81)*	24.66 *(1.23)*	28.42 *(1.41)*
	sh3_ova_50	20.94 *(1.60)*	19.49 *(0.68)*	25.92 *(3.84)*
	sh3_ova_300	33.82 *(1.39)*	25.77 *(2.17)*	30.26 *(2.49)*
	sh3_urea_50	14.34 *(0.38)*	15.09 *(0.05)*	15.24 *(0.24)*
	sh3_suc_300	17.28 *(0.12)*	17.18 *(0.16)*	17.57 *(0.21)*
GB1	gb1	14.20 *(0.40)*	14.59 *(0.10)*	14.53 *(0.09)*
	sh3_gb1_50	15.62 *(0.29)*	16.21 *(0.29)*	17.36 *(0.36)*
	sh3_gb1_100	19.06 *(0.34)*	19.23 *(0.62)*	21.65 *(0.58)*
	sh3_gb1_300	32.10 *(0.15)*	25.31 *(0.81)*	26.42 *(0.22)*
Lysozyme	lys	18.40 *(0.50)*	19.42 *(0.08)*	19.65 *(0.17)*
	sh3_lys_50	23.12 *(0.47)*	26.50 *(1.04)*	28.04 *(0.74)*
	sh3_lys_200	30.82 *(0.13)*	33.88 *(1.33)*	38.47 *(0.74)*
	sh3_lys_300	32.90 *(0.06)*	31.62 *(0.17)*	36.31 *(0.36)*
BSA	bsa	32.81 *(0.72)*	36.84 *(1.33)*	36.98 *(1.01)*
	sh3_bsa_50	37.71 *(0.33)*	41.92 *(1.25)*	45.19 *(0.17)*
	sh3_bsa_100	33.79 *(0.31)*	41.11 *(1.24)*	42.47 *(0.14)*
	sh3_bsa_200	32.61 *(0.09)*	42.38 *(1.00)*	44.59 *(0.47)*
	sh3_bsa_300	39.14 *(0.04)*	44.21 *(3.28)*	38.53 *(0.25)*
Ovalbumin	ova	33.98 *(0.08)*	37.52 *(1.13)*	37.63 *(0.82)*
	sh3_ova_50	34.47 *(0.80)*	34.80 *(1.58)*	36.88 *(0.23)*
	sh3_ova_300	38.50 *(0.06)*	44.60 *(1.71)*	39.05 *(0.32)*

[a]obtained from translational diffusion according to $R_h=k_BT/(6\pi\eta D_t)$
[b]obtained from rotational diffusion according to $R_h=(k_BT/(8\pi\eta D_r))^{1/3}$

Table S14. Fitting coefficients for translational and rotational diffusion from simulation to cluster model

Diffuser	Crowder	Colloid model (Eq. 20)				Mooney (Eq. 21)			
		Translation		Rotation		Translation		Rotation	
		ζ	χ^2	ζ	χ^2	ζ	χ^2	ζ	χ^2
SH3	GB1	27.24	*0.04*	16.03	*0.49*	18.81	*0.16*	13.56	*0.03*
	Lysozyme	95.07	*0.26*	89.56	*1.92*	73.29	*0.11*	81.20	*1.66*
	BSA	16.31	*0.76*	3.95	*68.4*	11.73	*1.87*	3.28	*102.5*
	Ovalbumin	71.36	*0.02*	31.20	*1.09*	52.12	*0.00*	27.03	*0.84*
GB1	GB1	31.27	*0.14*	17.50	*4.94*	22.15	*0.39*	15.03	*8.28*
Lysozyme	Lysozyme	42.72	*0.15*	46.20	*0.74*	31.29	*0.06*	41.05	*0.59*
BSA	BSA	10.00	*1.50*	10.54	*0.39*	4.97	*0.85*	8.32	*0.28*
Ovalbumin	Ovalbumin	9.98	*0.07*	0.04	*0.23*	3.60	*0.01*	-0.33	*0.42*

fit of simulation data against **Eq. 22** for translational diffusion or **Eq. 23** for rotational diffusion with different estimates of relative viscosities using the coefficients for MD simulations from **Table S8**.

Table S15. Translational diffusion coefficients of water from simulations

Protein	System	Translational Diffusion [Å²/ns]		
		$D_{t,PBC}$[a]	$D_{t,TIP3P}$[b]	$D_{t,MD}$[c]
SH3	sh3	520.4 *(0.3)*	544.0 *(0.3)*	204.2 *(0.1)*
	sh3_gb1_50	516.8 *(0.1)*	529.9 *(0.1)*	198.9 *(0.1)*
	sh3_gb1_100	469.4 *(0.5)*	479.4 *(0.5)*	179.9 *(0.2)*
	sh3_gb1_300	274.6 *(1.3)*	277.8 *(1.3)*	104.2 *(0.5)*
	sh3_lys_50	533.7 *(0.0)*	546.0 *(0.0)*	204.9 *(0.0)*
	sh3_lys_200	401.2 *(1.7)*	406.9 *(1.7)*	152.7 *(0.6)*
	sh3_lys_300	317.7 *(2.5)*	321.4 *(2.5)*	120.6 *(0.9)*
	sh3_bsa_50	538.8 *(0.0)*	547.0 *(0.0)*	205.3 *(0.2)*
	sh3_bsa_100	492.2 *(0.7)*	500.4 *(0.7)*	187.8 *(0.3)*
	sh3_bsa_200	398.7 *(2.0)*	405.0 *(2.0)*	152.0 *(0.8)*
	sh3_bsa_300	308.4 *(1.5)*	311.4 *(1.5)*	116.9 *(0.6)*
	sh3_ova_50	540.6 *(0.3)*	550.3 *(0.3)*	206.5 *(0.1)*
	sh3_ova_300	328.4 *(1.9)*	331.5 *(1.9)*	124.4 *(0.7)*
	sh3_urea_50	536.5 *(0.1)*	551.9 *(0.1)*	207.1 *(0.0)*
	sh3_suc_300	308.6 *(0.1)*	314.2 *(0.1)*	117.9 *(0.0)*
GB1	gb1	521.5 *(0.1)*	545.8 *(0.2)*	204.8 *(0.1)*
Lysozyme	lys	512.0 *(0.5)*	531.9 *(0.5)*	199.6 *(0.2)*
BSA	bsa	520.7 *(0.4)*	531.4 *(0.4)*	199.4 *(0.2)*
Ovalbumin	ova	495.8 *(0.3)*	506.2 *(0.3)*	190.0 *(0.1)*
	wat_salt[d]	568.8 (*0.3*)	606.4 *(0.3)*	227.6 *(0.1)*
	wat[d]	584.9 *(0.4)*	622.5 *(0.4)*	233.6 *(0.1)*

[a]estimated directly from MSD curves according to **Eq. 10** over 2-10 ns
[b]corrected for periodic box artefacts according to **Eq. 11**
[c]corrected for underestimated viscosity with TIP3P water model according to **Eq. 13**
[d]diffusion was estimated by averaging over 150 simulations of 1 ns each

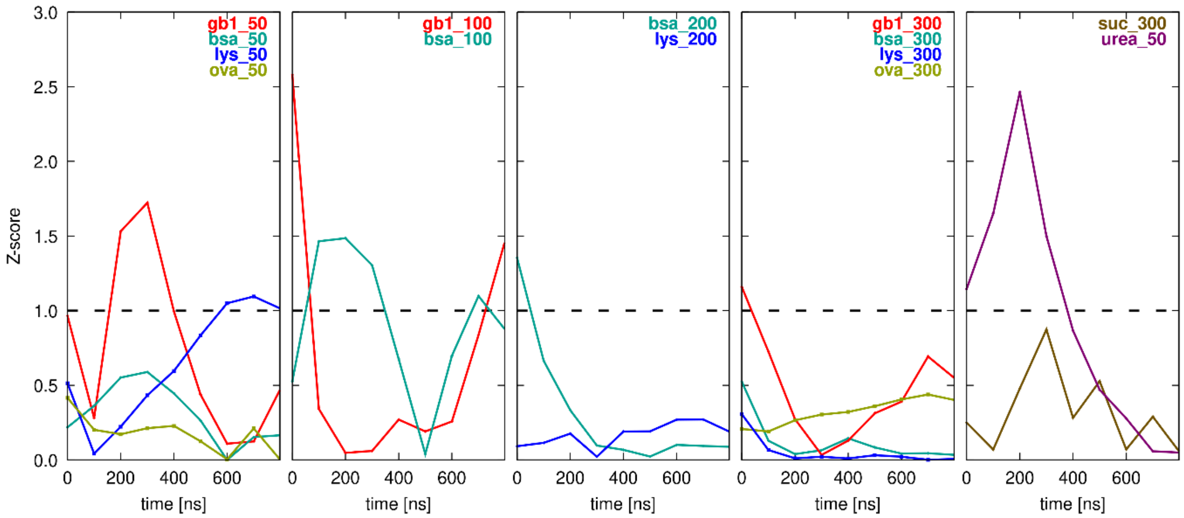

Figure S1. Simulation convergence. Based on average number of interactions with crowders a Z-score is shown as a function of simulation time t comparing the average over [t,t+400ns] (a_{400}) *vs.* the average over [t, t_{max}] (a_{max}), where t_{max} is the total simulation time used for analysis according to **Table S2**. The Z-score was calculated as $|a_{400} - a_{max}|/(SEM(a_{400}) + SEM(a_{max}))$ using the standard error of the mean (SEM) for the two averages. The dashed lines indicate a Z-score of 1.

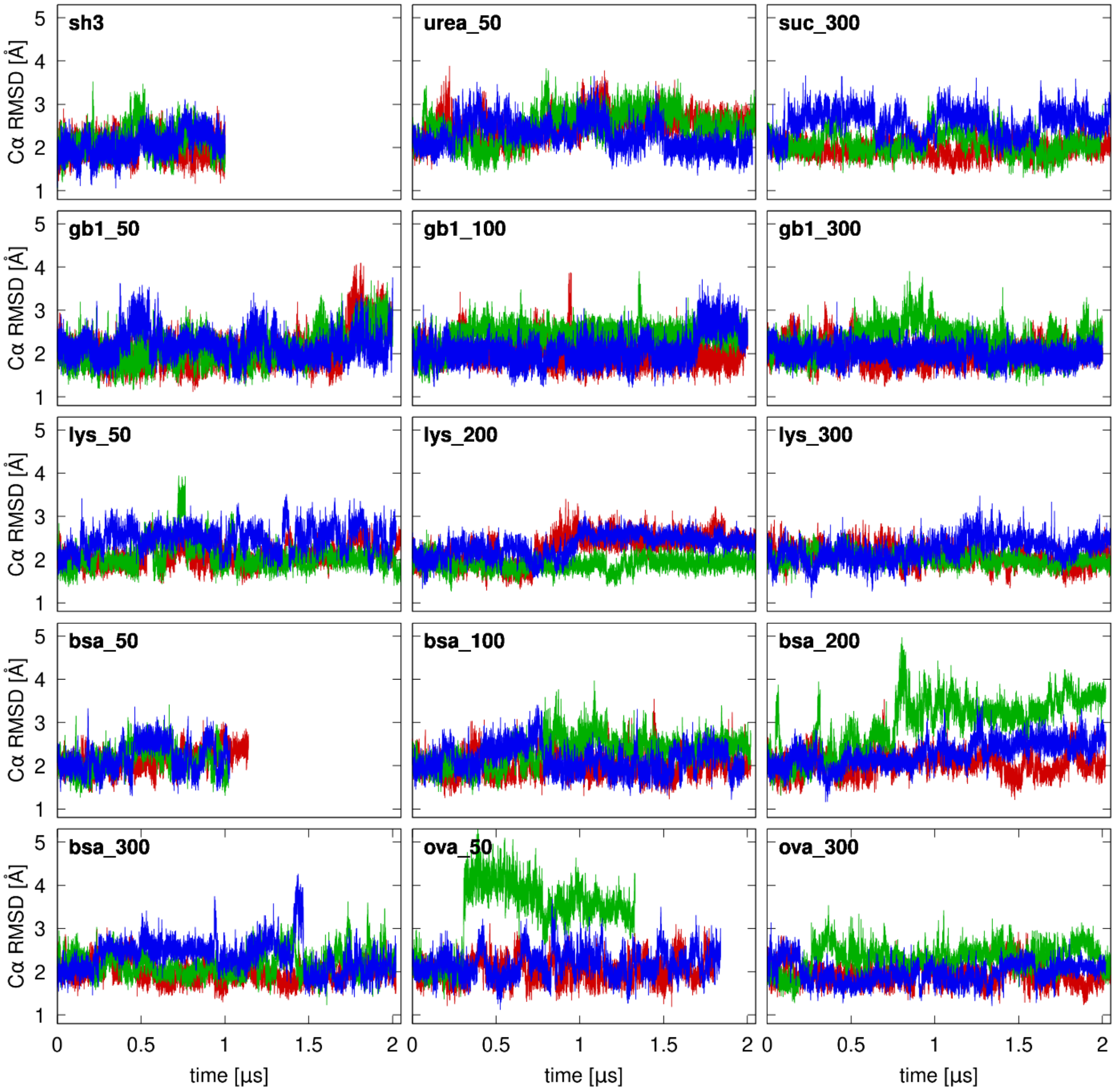

Figure S2. Coordinate root mean-square deviation time series for SH3. RMSD values were calculated for Cα atoms with respect to 2A37 after optimal superposition. Colors distinguish different simulation replicates.

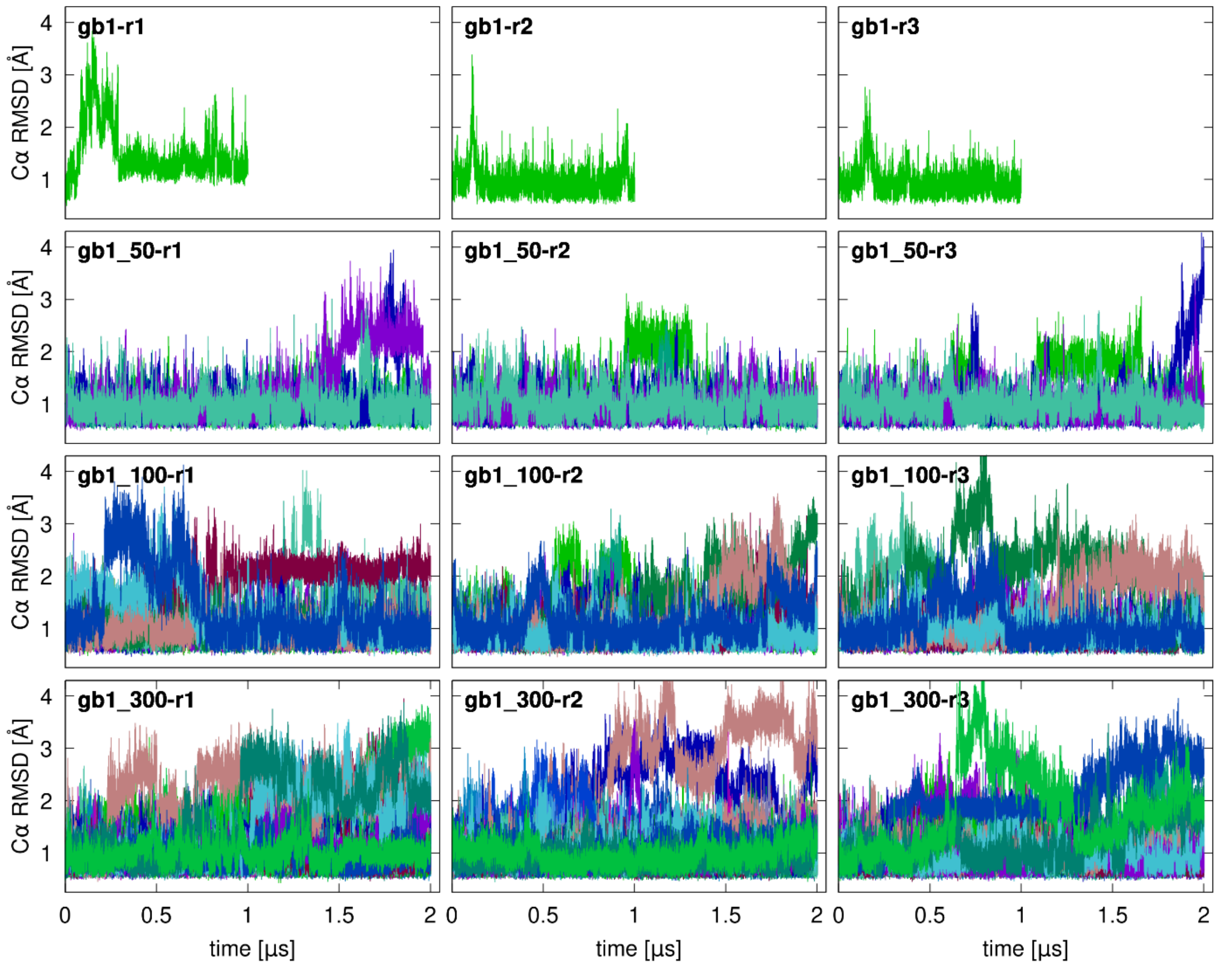

Figure S3. Coordinate root mean-square deviation time series for GB1. Results in columns 1-3 correspond to simulation replicates for each system. RMSD values were calculated for Cα atoms with respect to 2LGI after optimal superposition. Colors distinguish different protein copies.

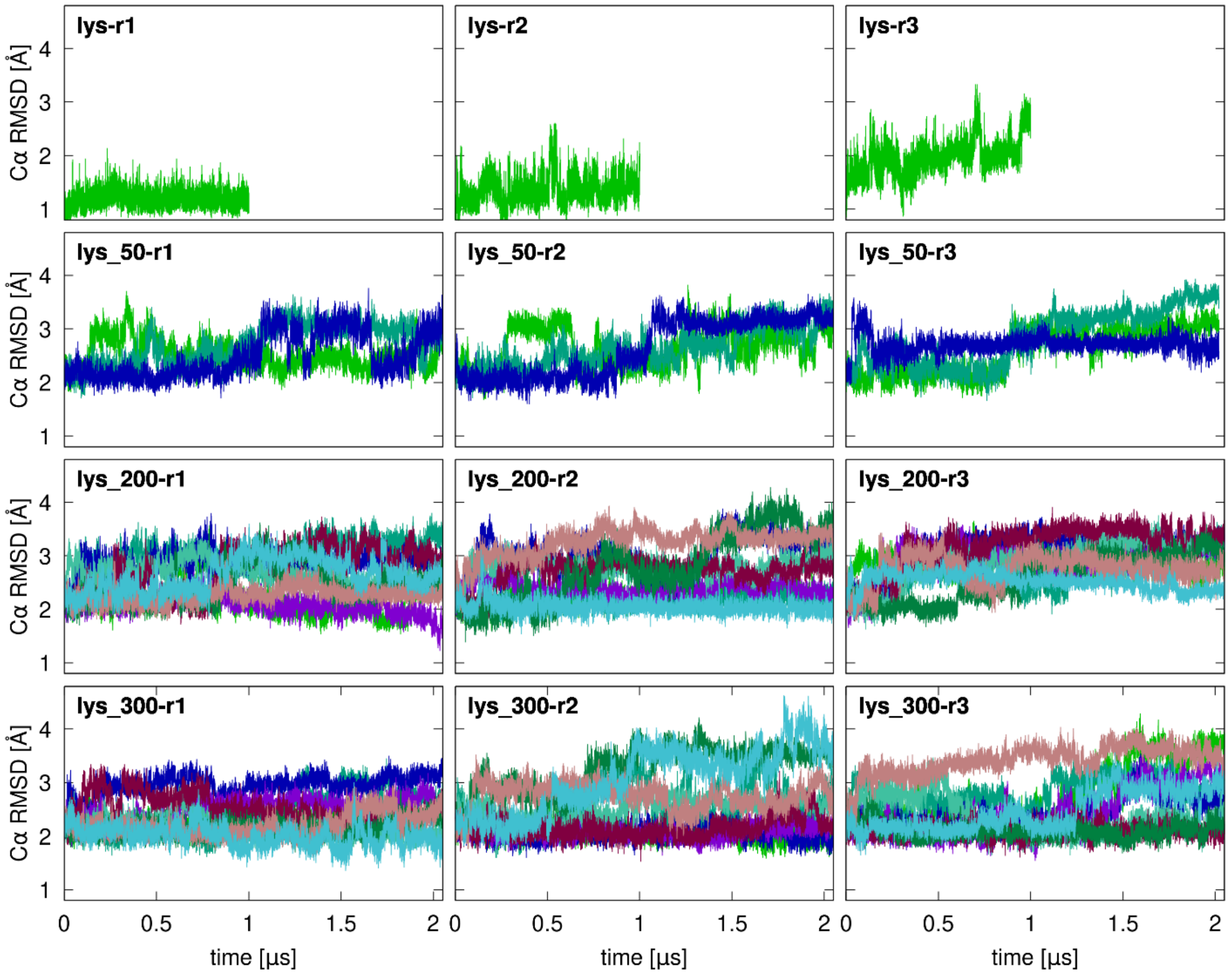

Figure S4. Coordinate root mean-square deviation time series for lysozyme. Results in columns 1-3 correspond to simulation replicates for each system. RMSD values were calculated for Cα atoms with respect to 2WL2 after optimal superposition. Colors distinguish different protein copies.

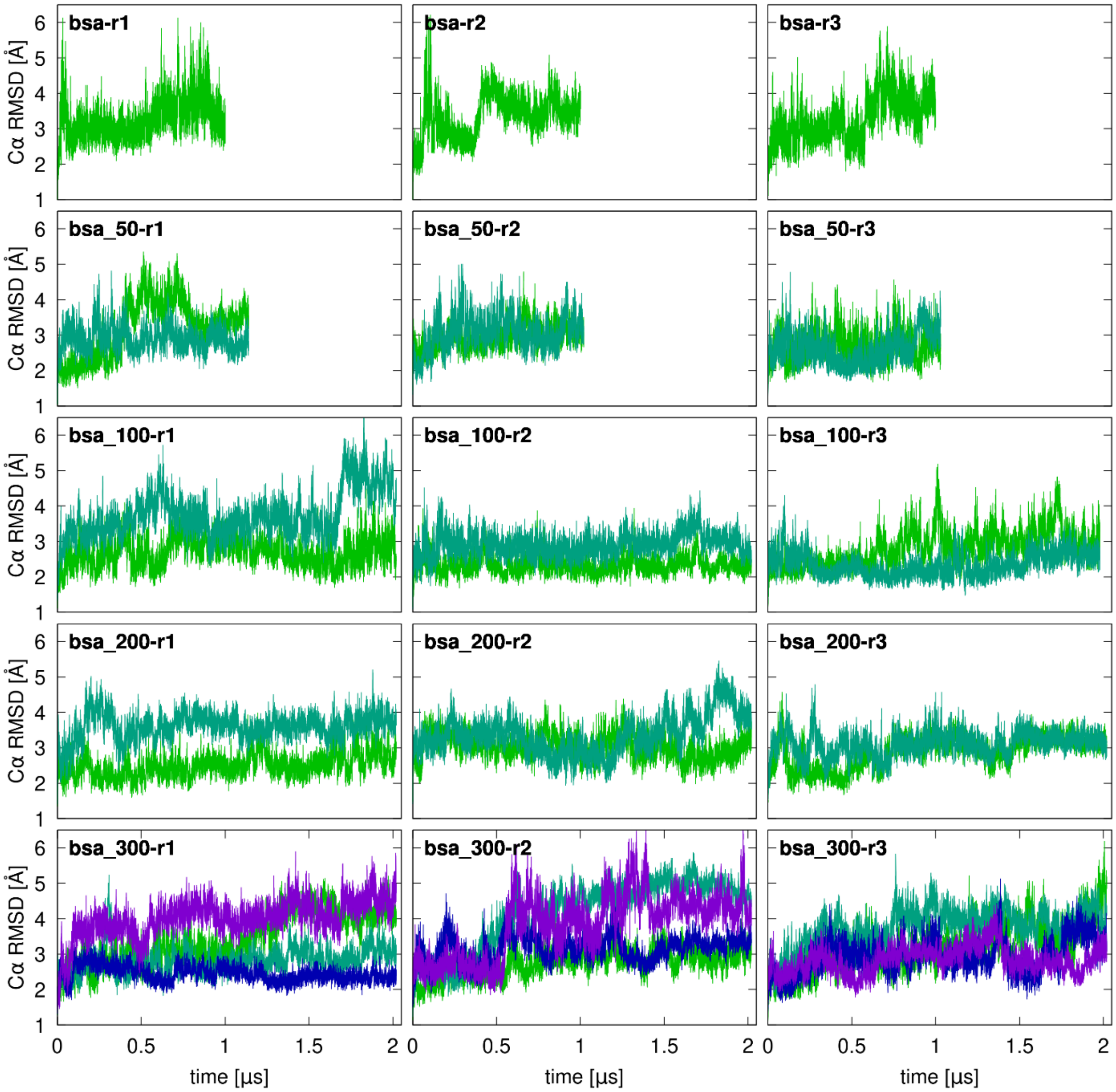

Figure S5. Coordinate root mean-square deviation time series for BSA. Results in columns 1-3 correspond to simulation replicates for each system. RMSD values were calculated for Cα atoms with respect to 3V03 after optimal superposition. Colors distinguish different protein copies.

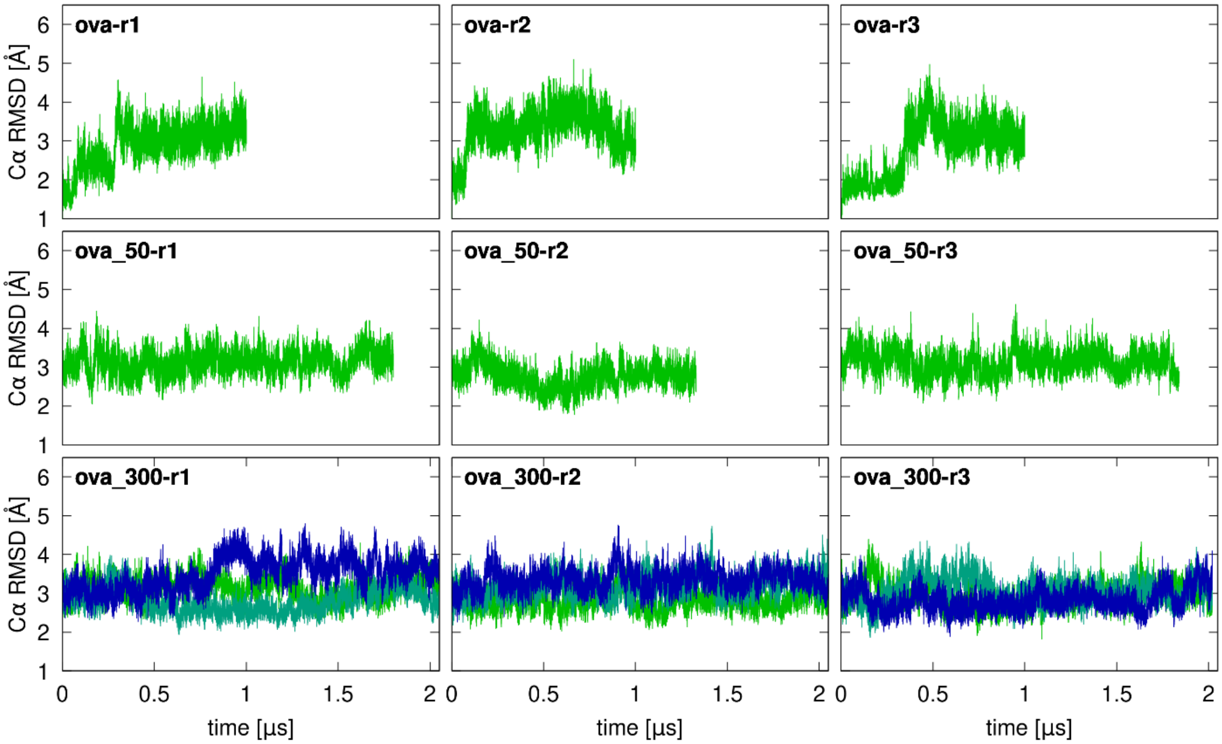

Figure S6. Coordinate root mean square deviation time series for ovalbumin. Results in columns 1-3 correspond to simulation replicates for each system. RMSD values were calculated for Cα atoms with respect to 1OVA after optimal superposition. Colors distinguish different protein copies. Note that ovalbumin was modeled as a homo-dimer.

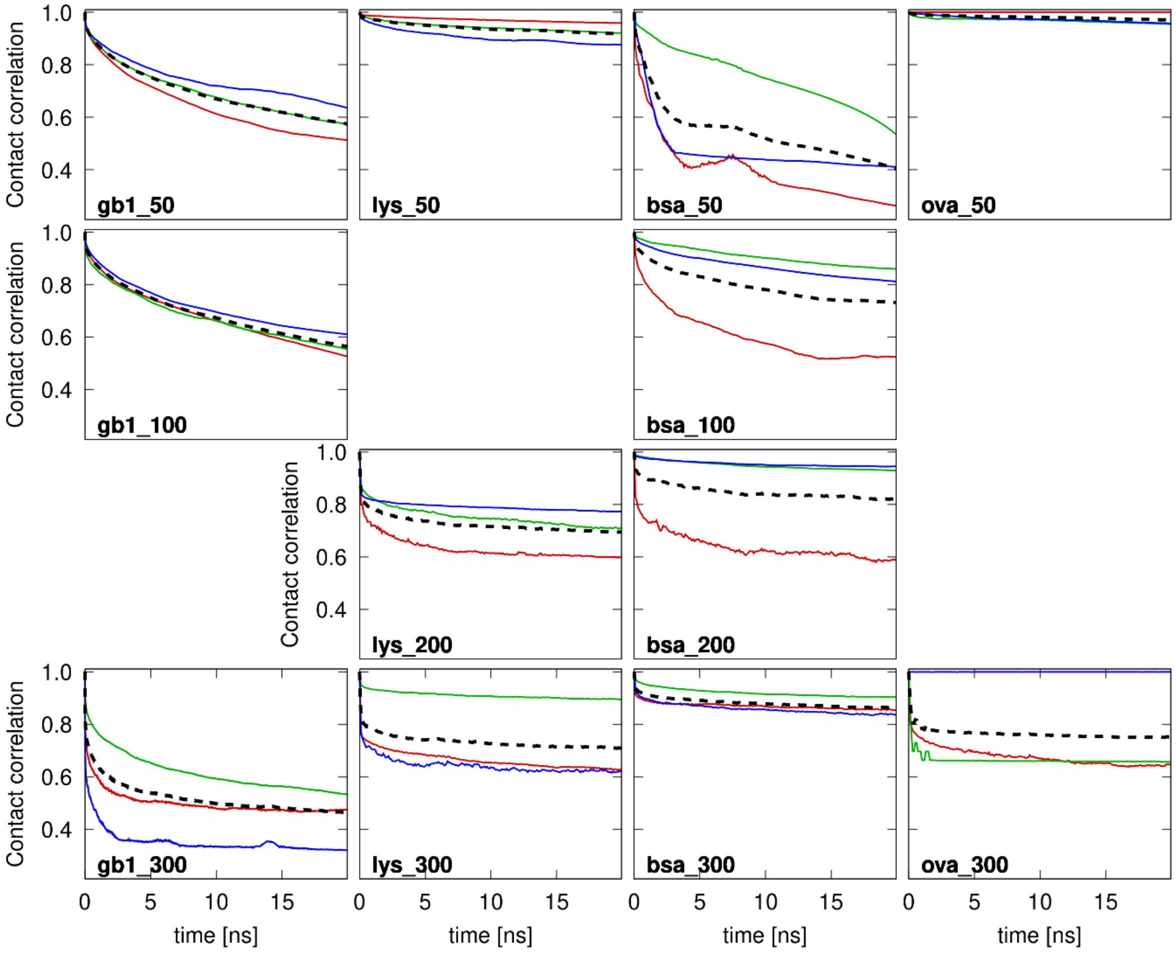

Figure S7. SH3-protein crowder contact survival functions. Contact survival functions between SH3 and protein crowder molecules were calculated according to **Eq. 2**. Survival functions averaged over crowder proteins for different replicates are shown in red, blue, and green. Averaged contact functions used for fitting are shown as black dashed lines. Fits are reported in **Table S5**.

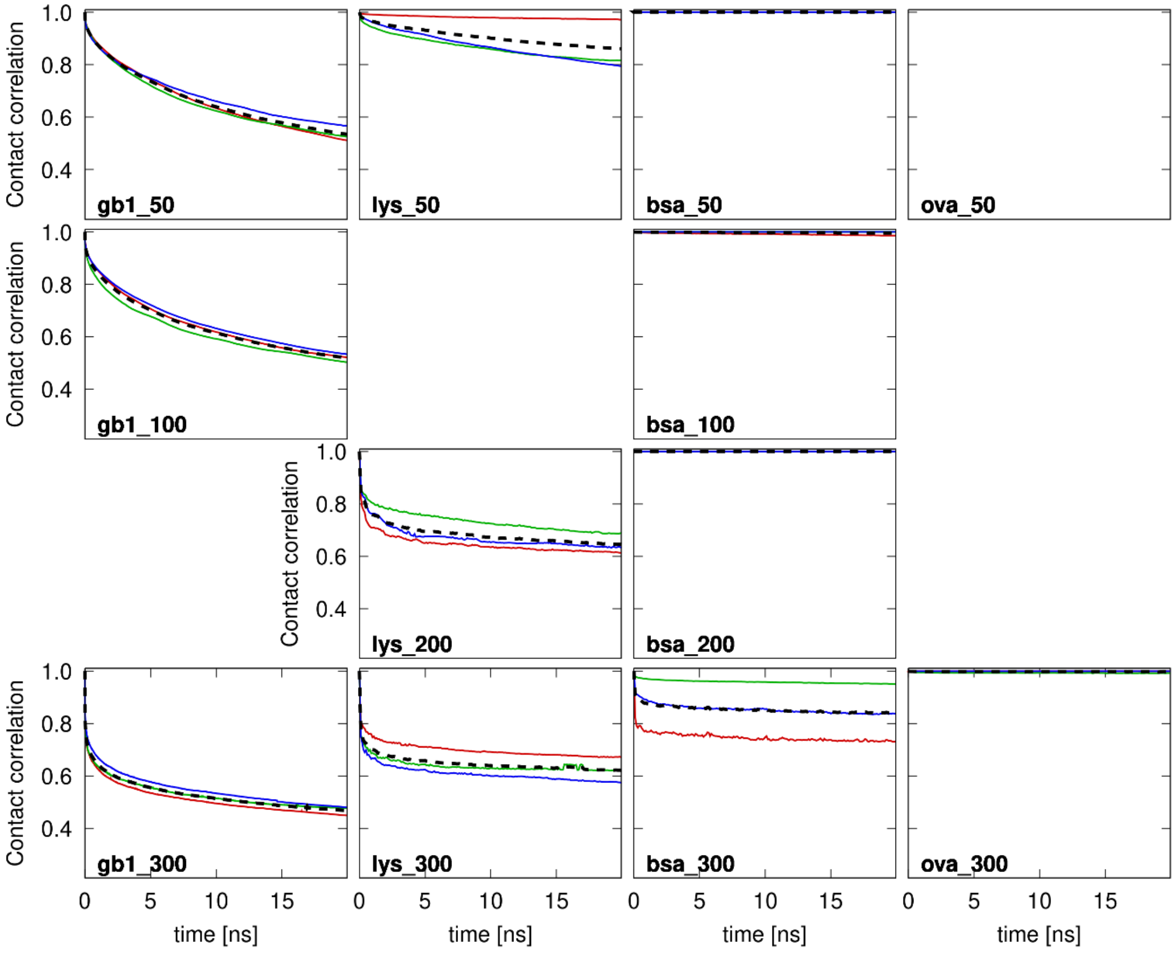

Figure S8. Crowder contact survival functions for systems with SH3. Contact survival functions were calculated according to **Eq. 2**. Survival functions averaged over crowders are shown in red, blue, and green for different replicates. Contact functions averaged over replicates that were used for fitting are shown as black dashed lines. Fits are reported in **Table S6**.

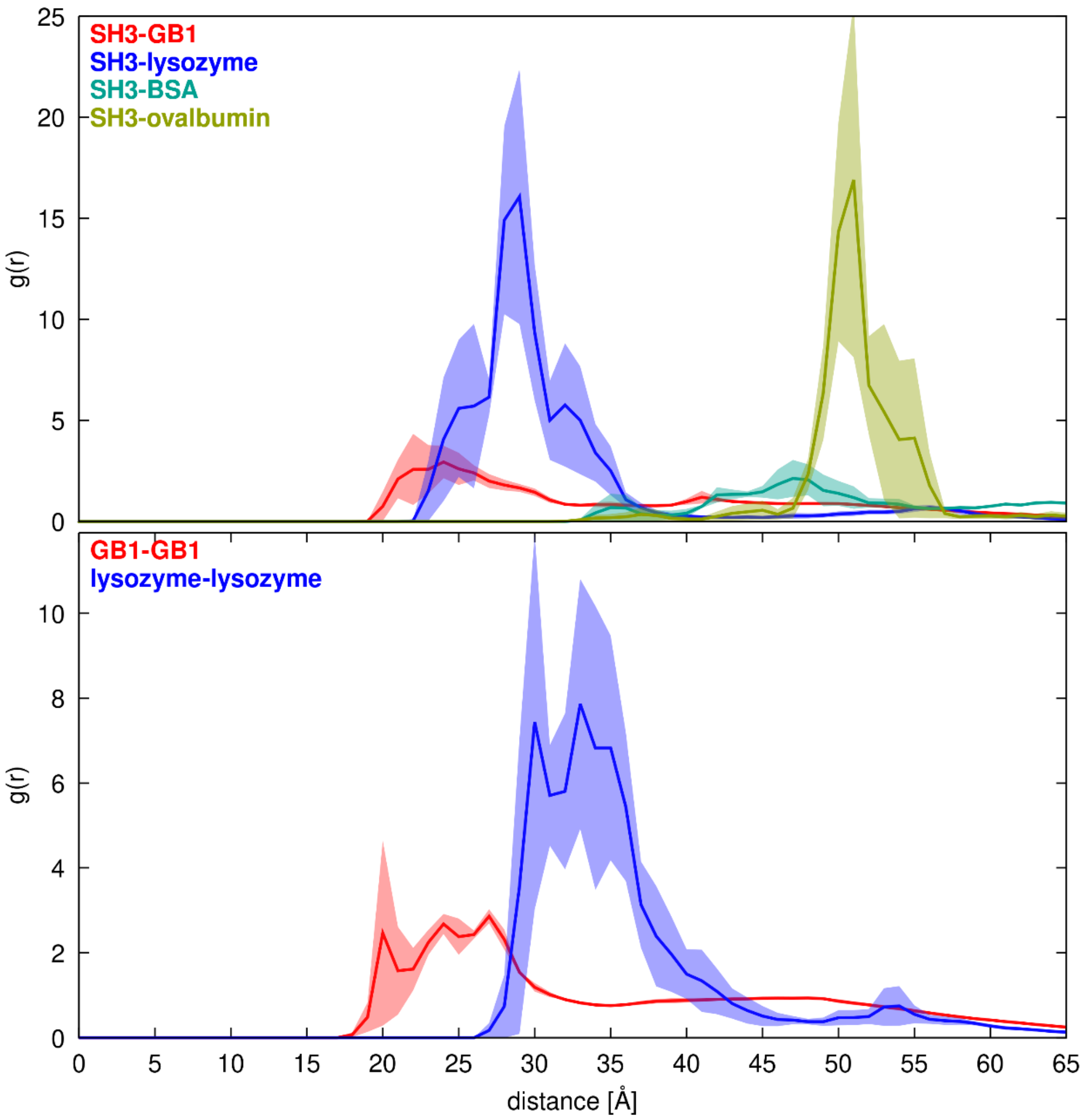

Figure S9. Protein-protein radial distribution functions. RDFs were calculated from protein centers of mass between SH3 and crowders (in *sh3_gb1_50*, *sh3_lys_50*, *sh3_bsa_50*, sh3_ova_50) and between GB1 or lysozyme crowders (in *sh3_gb1_50*, *sh3_lys_50*).

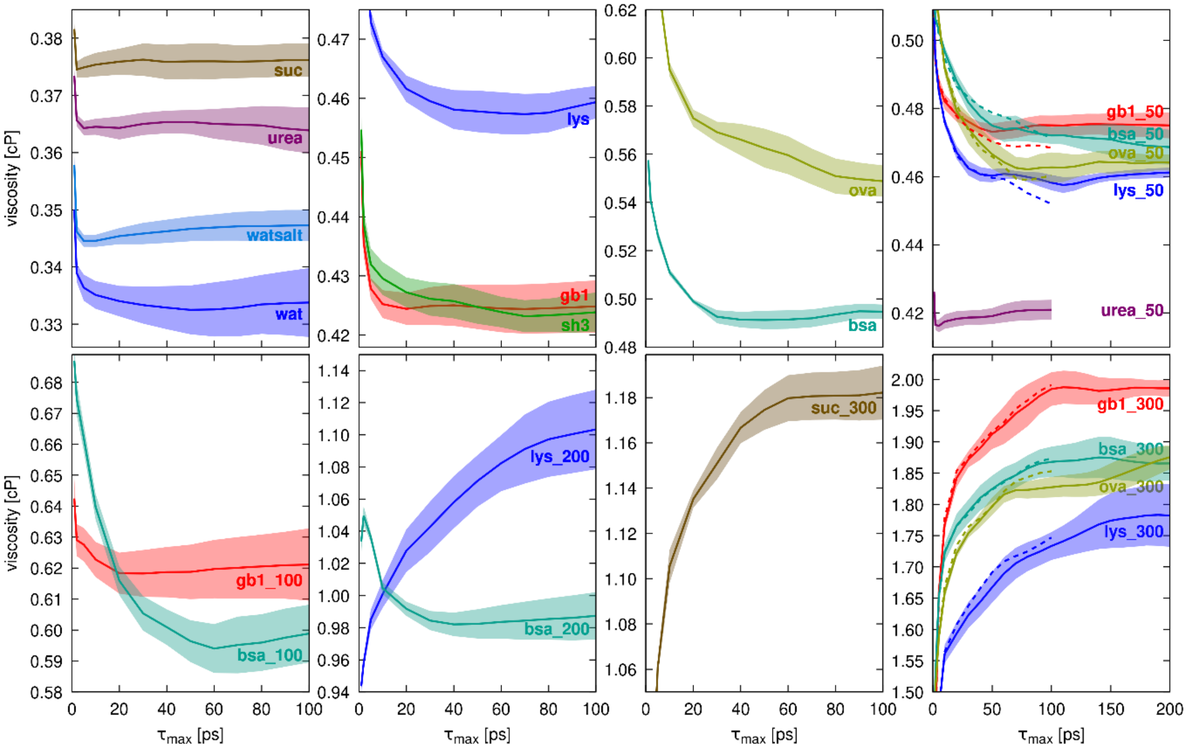

Figure S10. Convergence of viscosity estimates from simulations. Viscosities were estimated according to **Eq. 3** following the protocol by Zhang *et al.*[21] as a function of τ_{max}. For each estimate, pressure tensor fluctuation from 150 simulations over 1 or 2 ns were used. The shaded areas indicate the standard error from variations in viscosity estimates obtained from individual trajectories. For systems that were simulated over 2 ns, the dashed line indicates viscosity estimates obtained using only the first 1 ns.

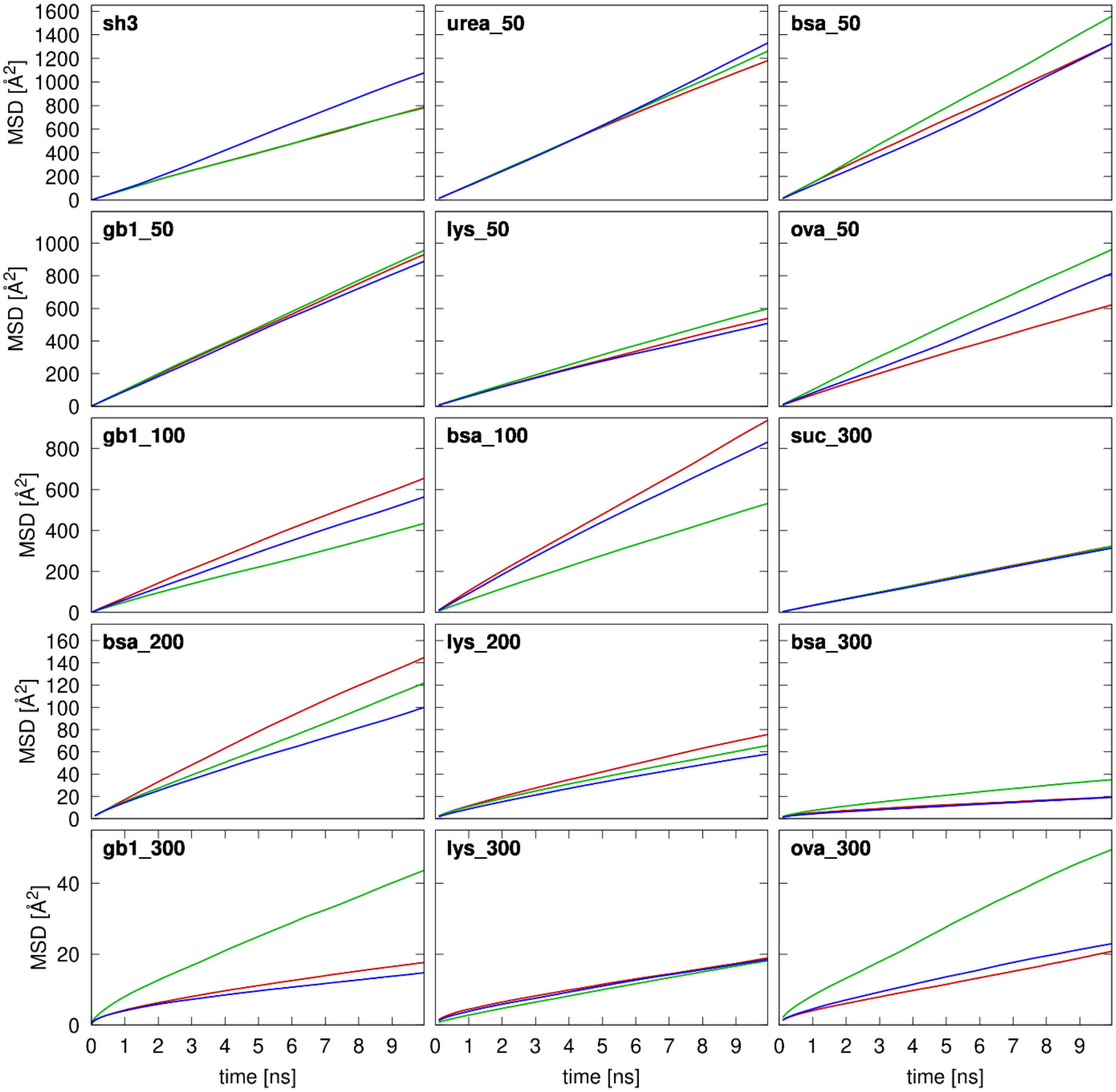

Figure S11. Mean-square displacement vs. time for SH3. Colors distinguish results from three replicates.

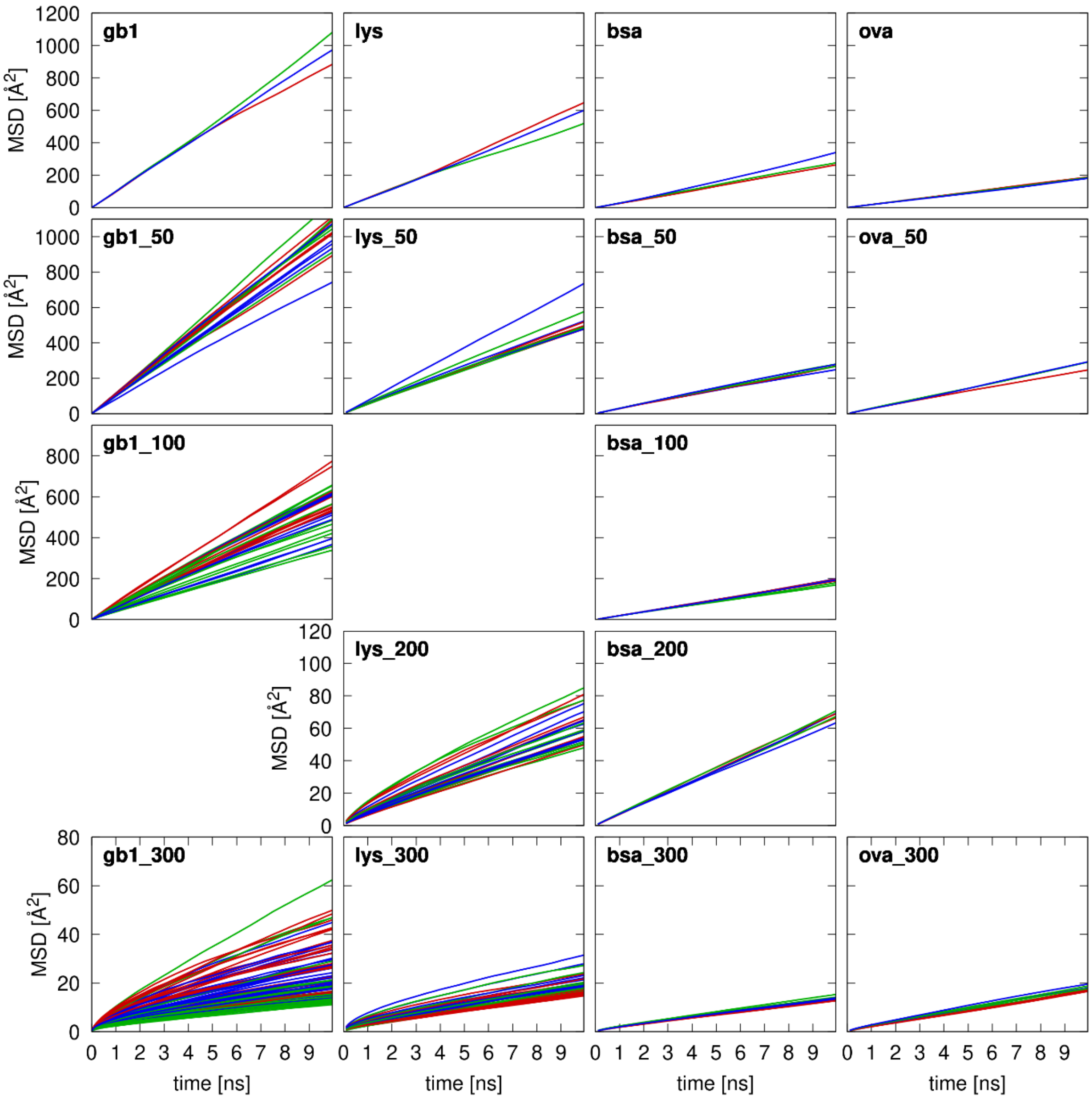

Figure S12. Mean-square displacement vs. time for crowder proteins in systems with SH3. Individual curves are for different protein copies. Colors distinguish results from three replicates.

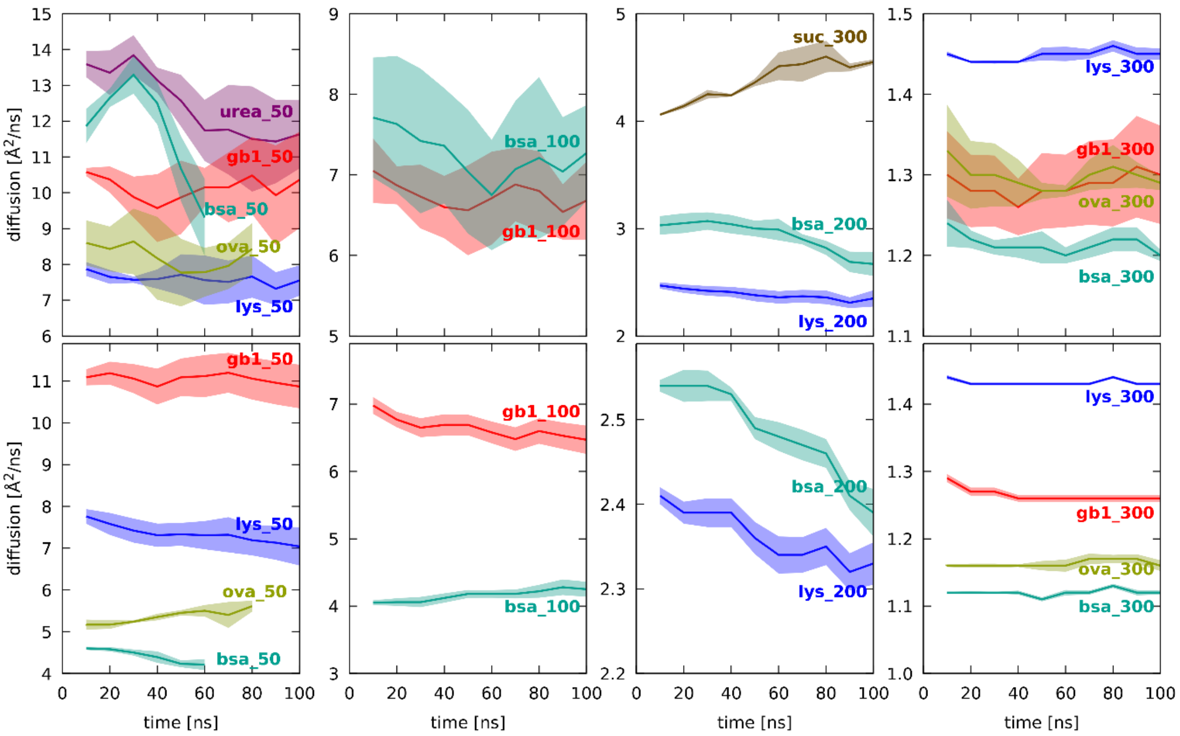

Figure S13. Translatonal diffusion from simulations vs. time. Diffusion constants were corrected for periodic boundaries and underestimated viscosity with TIP3P water. The time reflects the 10 ns time interval used for fitting MSD vs. time. Results for SH3 diffusion are shown in the top row, crowder diffusion is shown in the bottom.

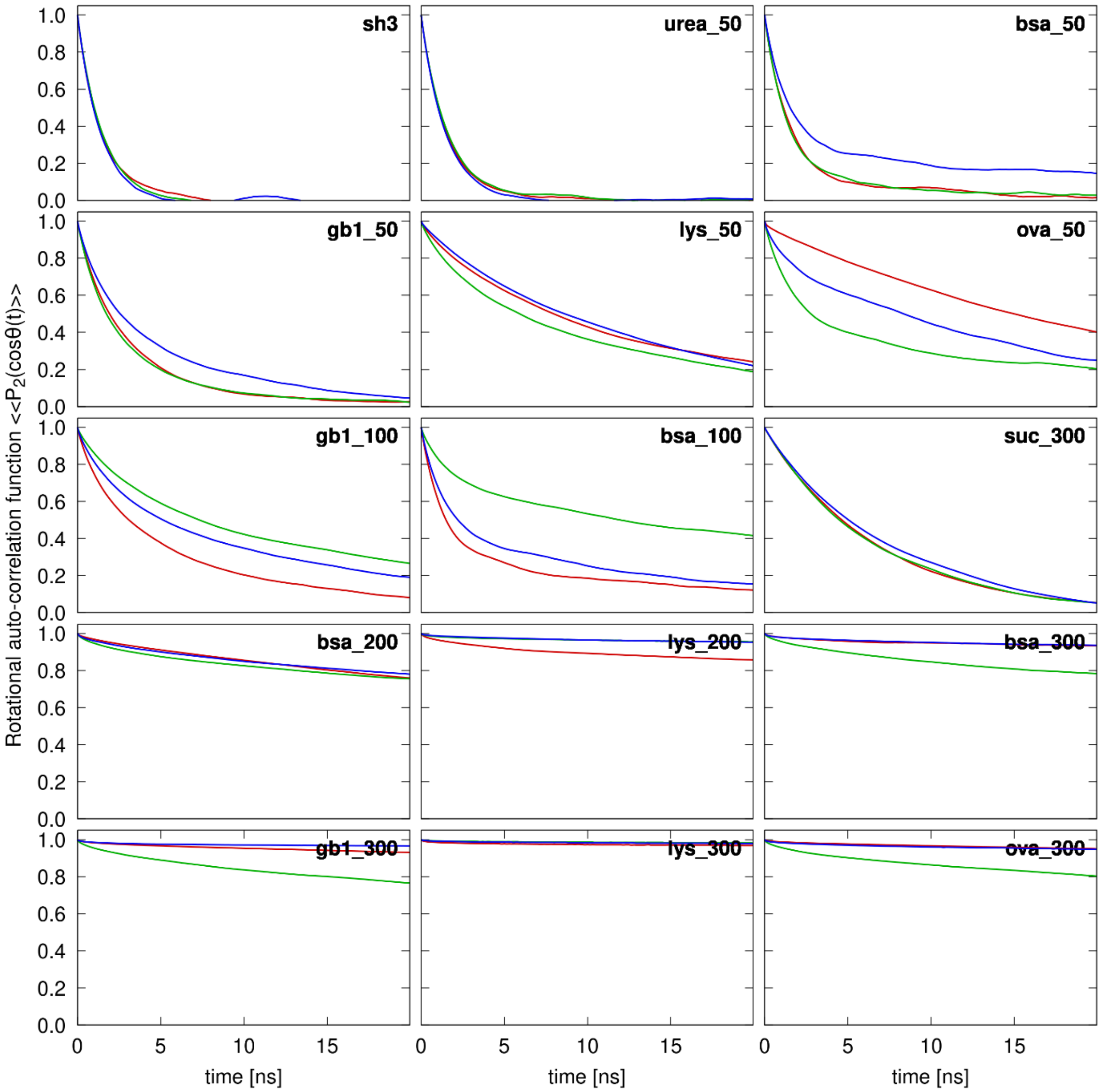

Figure S14. Rotational correlation functions for SH3. Colors distinguish results from three replicates.

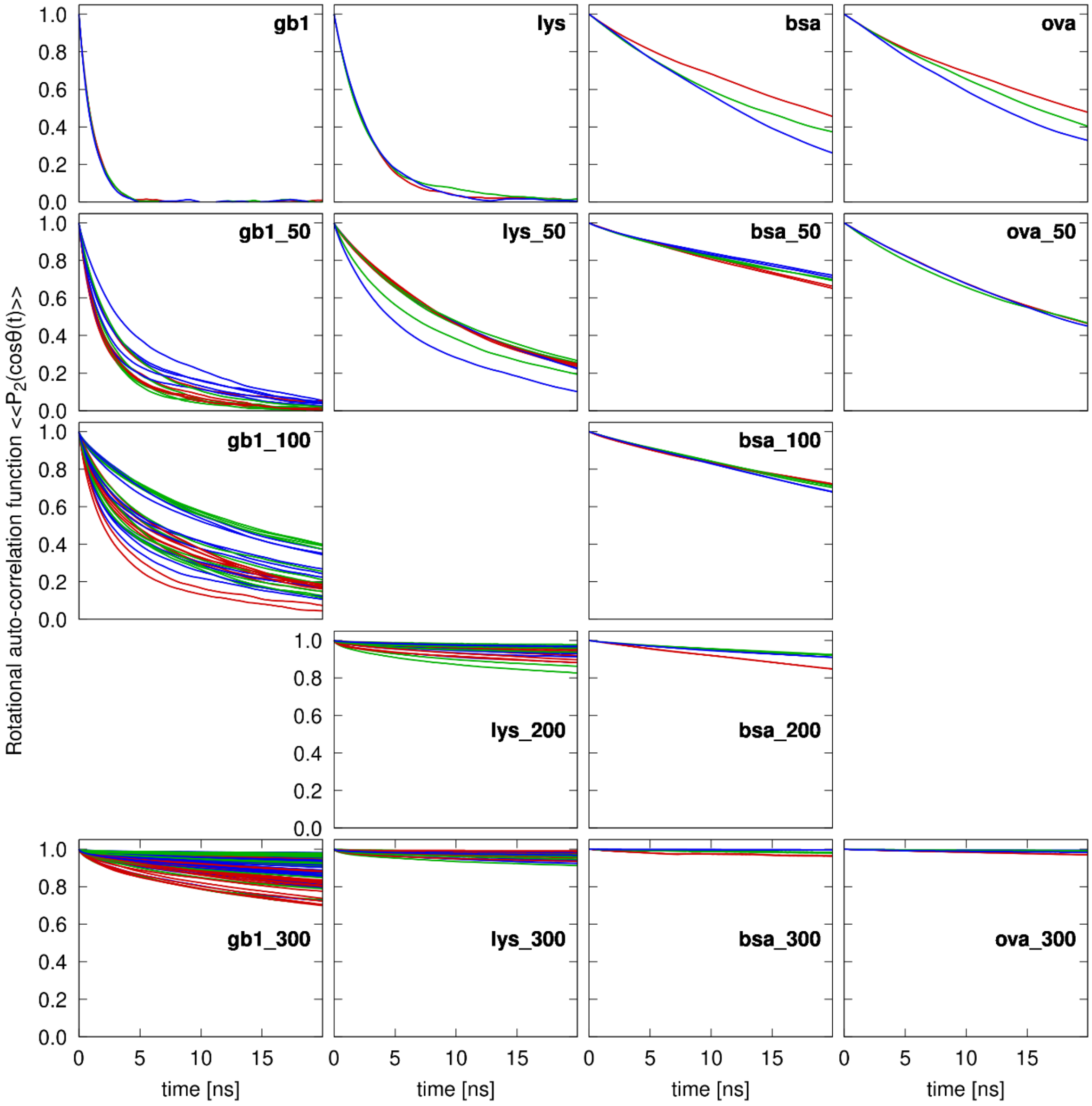

Figure S15. Rotational correlation functions for crowder proteins in systems with SH3. Individual curves are for different protein copies. Colors distinguish results from three replicates.

SUPPLEMENTARY REFERENCES

(1) Bezsonova, I.; Singer, A.; Choy, W.-Y.; Tollinger, M.; Forman-Kay, J. D. Structural Comparison of the Unstable drkN SH3 Domain and a Stable Mutant. *Biochemistry* **2005**, *44*, 15550-15560.
(2) Wylie, B. J.; Sperling, L. J.; Nieuwkoop, A. J.; Franks, W. T.; Oldfield, E.; Rienstra, C. M. Ultrahigh resolution protein structures using NMR chemical shift tensors. *Proc. Natl. Acad. Sci. U. S. A.* **2011**, *108*, 16974-16979.
(3) Majorek, K. A.; Porebski, P. J.; Dayal, A.; Zimmerman, M. D.; Jablonska, K.; Stewart, A. J.; Chruszcz, M.; Minor, W. Structural and immunologic characterization of bovine, horse, and rabbit serum albumins. *Mol. Immunol.* **2012**, *52*, 174-182.
(4) Stein, P. E.; Leslie, A. G. W.; Finch, J. T.; Carrell, R. W. Crystal structure of uncleaved ovalbumin at 1·95 Å resolution. *J. Mol. Biol.* **1991**, *221*, 941-959.
(5) Ortega, A.; Amorós, D.; De La Torre, J. G. Prediction of hydrodynamic and other solution properties of rigid proteins from atomic-and residue-level models. *Biophys. J.* **2011**, *101*, 892-898.
(6) Stadmiller, S. S.; Aguilar, J. S.; Parnham, S.; Pielak, G. J. Protein–Peptide Binding Energetics under Crowded Conditions. *J. Phys. Chem. B* **2020**, *124*, 9297-9309.
(7) Liu, Y.; Porcar, L.; Chen, J.; Chen, W.-R.; Falus, P.; Faraone, A.; Fratini, E.; Hong, K.; Baglioni, P. Lysozyme Protein Solution with an Intermediate Range Order Structure. *J. Phys. Chem. B* **2011**, *115*, 7238-7247.
(8) Mikol, V.; Hirsch, E.; Giegé, R. Diagnostic of precipitant for biomacromolecule crystallization by quasi-elastic light-scattering. *J. Mol. Biol.* **1990**, *213*, 187-195.
(9) Altieri, A. S.; Hinton, D. P.; Byrd, R. A. Association of Biomolecular Systems via Pulsed Field Gradient NMR Self-Diffusion Measurements. *J. Am. Chem. Soc.* **1995**, *117*, 7566-7567.
(10) Coffman, J. L.; Lightfoot, E. N.; Root, T. W. Protein diffusion in porous chromatographic media studied by proton and fluorine PFG-NMR. *J. Phys. Chem. B* **1997**, *101*, 2218-2223.
(11) Meechai, N.; Jamieson, A. M.; Blackwell, J. Translational diffusion coefficients of bovine serum albumin in aqueous solution at high ionic strength. *J. Colloid Interface Sci.* **1999**, *218*, 167-175.
(12) Gaigalas, A. K.; Hubbard, J. B.; McCurley, M.; Woo, S. Diffusion of bovine serum albumin in aqueous solutions. *J. Phys. Chem.* **1992**, *96*, 2355-2359.
(13) Gibbs, S. J.; Chu, A. S.; Lightfoot, E. N.; Root, T. W. Ovalbumin diffusion at low ionic strength. *J. Phys. Chem.* **1991**, *95*, 467-471.
(14) Wong, V.; Case, D. A. Evaluating rotational diffusion from protein MD simulations. *J. Phys. Chem. B* **2008**, *112*, 6013-6024.
(15) Linke, M.; Köfinger, J.; Hummer, G. Fully Anisotropic Rotational Diffusion Tensor from Molecular Dynamics Simulations. *J. Phys. Chem. B* **2018**, *122*, 5630-5639.
(16) Shapiro, Y. E.; Meirovitch, E. Slowly Relaxing Local Structure (SRLS) Analysis of 15N–H Relaxation from the Prototypical Small Proteins GB1 and GB3. *J. Phys. Chem. B* **2012**, *116*, 4056-4068.
(17) Idiyatullin, D.; Nesmelova, I.; Daragan, V. A.; Mayo, K. H. Heat Capacities and a Snapshot of the Energy Landscape in Protein GB1 from the Pre-denaturation Temperature Dependence of Backbone NH Nanosecond Fluctuations. *J. Mol. Biol.* **2003**, *325*, 149-162.
(18) Gottschalk, M.; Halle, B. Self-Association of Lysozyme as Seen by Magnetic Relaxation Dispersion. *J. Phys. Chem. B* **2003**, *107*, 7914-7922.
(19) Buck, M.; Boyd, J.; Redfield, C.; Mackenzie, D. A.; Jeenes, D. J.; Archer, D. B.; Dobson, C. M. Structural determinants of protein dynamics: analysis of 15N NMR relaxation measurements for main-chain and side-chain nuclei of hen egg white lysozyme. *Biochemistry* **1995**, *34*, 4041-4055.
(20) Wang, R.; Bright, F. V. Effects of protein denaturation on the rotational reorientation kinetics of dansylated bovine serum albumin. *J. Phys. Chem.* **1993**, *97*, 10872-10878.
(21) Zhang, Y.; Otani, A.; Maginn, E. J. Reliable Viscosity Calculation from Equilibrium Molecular Dynamics Simulations: A Time Decomposition Method. *J. Chem. Theory Comput.* **2015**, *11*, 3537-3546.
